# Sexual Orientation and Sex Differences in Adult Chronic Conditions, Health Risk Factors, and Protective Health Practices, Oregon, 2005–2008

**DOI:** 10.5888/pcd11.140126

**Published:** 2014-08-07

**Authors:** Rodney Y. Garland-Forshee, Steven C. Fiala, Duyen L. Ngo, Katarina Moseley

**Affiliations:** Author Affiliations: Steven C. Fiala, Duyen L. Ngo, Katarina Moseley, Oregon Health Authority, Portland, Oregon.

## Abstract

**Introduction:**

Research on lesbian, gay, and bisexual (LGB) individuals’ health and health practices has primarily consisted of convenience studies focused on HIV/AIDS, substance use, or mental illness. We examined health-related disparities among Oregon LGB men and women compared with heterosexual men and women using data from a population-based survey.

**Methods:**

Data from the 2005 through 2008 Oregon Behavioral Risk Factor Surveillance System were used to examine associations between sexual orientation and chronic conditions, health limitations, health risk factors, and protective health practices.

**Results:**

Compared with heterosexual women, lesbian and bisexual women were significantly more likely to smoke cigarettes, be obese, binge drink, and have chronic conditions, and less likely to engage in protective health practices. Compared with heterosexual men, gay men were significantly less likely to be obese, more likely to binge drink, and more likely to engage in protective health practices. Compared with heterosexual men, bisexual men were significantly more likely to have a physical disability, smoke cigarettes, binge drink, and more likely to get an HIV test.

**Conclusions:**

Health disparities among Oregon LGB individuals were most prominent among lesbian and bisexual women. Gay men had the most protective health practices, but they were more likely than heterosexual men to engage in risky behaviors that lead to chronic diseases later in life. Targeted public health interventions should be provided in environments that avoid stigmatizing and discriminating against LGB individuals where they live, work, learn, and socialize.

## Introduction

Research on lesbian, gay, and bisexual (LGB) individuals’ health and health practices has primarily consisted of convenience studies focused on HIV/AIDS, substance use, or mental illness (1,2). Recently, population-based studies have assessed chronic conditions, health risk factors, disabilities, physical or mental health limitations, and health practices of the adult LGB population (3,4). These and other studies reported that lesbian or bisexual women had higher odds of having arthritis (5), asthma (3,4), and diabetes (4), and were more likely to smoke cigarettes (3,4), abuse alcohol (3), and be overweight compared with heterosexual women (4). Lesbian and bisexual women were also more likely than heterosexual women to report poor health (6) and were less likely to receive preventive screenings (4,7). Compared with heterosexual men, gay or bisexual men had higher odds of having asthma (3), were more likely to smoke cigarettes (3,4), and were more likely to abuse alcohol (8). 

To understand the health of LGB adults in Oregon, the Oregon Public Health Division added a consistent sexual orientation question to the state’s Behavioral Risk Factor Surveillance System (BRFSS) survey in 2005. Addition of sexual orientation questions to state and federal surveys is a key recommendation of the 2011 Institute of Medicine (IOM) report on the health of LGB and transgender people and is an objective of *Healthy People 2020* (2,9). The IOM acknowledges that there is incomplete information about the health of LGB people, a group that is becoming increasingly visible and more socially acknowledged (2). This study addresses gaps in the literature by assessing additional chronic disease risk factors and preventive health behaviors and by controlling for additional demographic factors that are known to shape LGB health outcomes.

## Methods

Oregon BRFSS data from 2005 through 2008 were combined to provide a sufficient sample of LGB individuals for analysis. The BRFSS is a population-based telephone survey of Oregon adults conducted in collaboration with the Centers for Disease Control and Prevention (CDC). Data were collected from random-digit–dialed landline telephones with 1 adult respondent randomly selected from each household. Eligible respondents were noninstitutionalized English- or Spanish-speaking adults. All data have sample weights based on selection probability of a respondent and were poststratified to the age and sex characteristics of the Oregon population. All measures used for analysis were based on questions developed by CDC, except for the question on a respondent’s sexual orientation; CDC did not have a sexual orientation question at the time of survey administration.

### Measures

**Sexual orientation.** Starting in 2005, a consistent question on sexual orientation was added to the Oregon BRFSS. Survey respondents were asked, “Now I’m going to ask you a question about sexual orientation. Do you consider yourself to be a) heterosexual, that is, straight; b) homosexual, that is gay or lesbian; c) bisexual; or d) other?” For this study, the sexual orientation question was combined with the respondent’s sex to create the classification groups of gay, bisexual, and heterosexual men and lesbian, bisexual, and heterosexual women. From 2005 through 2008, 44,560 adult Oregonians were asked their sexual orientation on the Oregon BRFSS. A small group (n = 1,814; 4.1% unweighted) did not provide their sexual orientation, restricting the sample to 42,746 adult Oregonians.

**Demographics.** All demographic variables were recoded for analysis. Respondent age (in years) was recoded into 3 groups: 18 to 34, 35 to 54, and 55 years or older. Relationship status was combined into married or a member of an unmarried couple, formerly married, or never married. Unmarried couples were grouped with married respondents because, in Oregon, LGB couples could not marry at the time of survey administration. Educational attainment was grouped into 3 categories: high school degree or GED (general educational development) or less, attended 1 to 3 years of college, or attended 4 or more years of college. Rural or urban residency was determined by using zip codes classified by Rural-Urban Commuting Area (RUCA) codes (10). Urban areas had RUCA codes of 1.0, 1.1, 2.0, 2.1, 3.0, 4.1, 5.1, 7.1, 8.1, or 10.1; rural areas had codes of 4.0, 4.2, 5.0, 5.2, 6.0, 6.1, 7.0, 7.2, 7.3, 7.4, 8.0, 8.2, 8.3, 8.4, 9.0, 9.1, 9.2, 10.0, 10.2, 10.3, 10.4, 10.5, or 10.6 (10). Employment status was categorized as employed, unemployed or unable to work, or not in the workforce. Annual household income was categorized as less than $20,000, $20,000 to $49,999, and $50,000 or more. Because of the limited sample of nonwhite and Latino respondents, all nonwhite and Latino Oregonians were combined for comparison to white non-Latinos.

**Chronic conditions and health limitations.** Chronic conditions assessed included the following: asthma, arthritis, diabetes, high blood pressure, high cholesterol, and cardiovascular disease. Cardiovascular disease questions measured respondents who had ever had a heart attack, angina or coronary heart disease, or a stroke. Two questions on disabilities asked respondents if they used any special equipment and if they were limited in any activities. Respondents who reported activity limitations of 7 or more days during the last 30 days because of poor mental or physical health were assigned to the poor mental/physical health category.

**Health risk factors.** Current cigarette smokers were defined as having smoked 100 or more cigarettes in their lifetime and currently smoking cigarettes on some or all days. Obesity was established for respondents who had a body mass index (weight in kilograms divided by the square of height in meters) of 30 kg/m^2^ or higher. CDC defines heavy drinking as consuming more than 1 drink per day on average in the last month for women and consuming more than 2 drinks per day on average in the last month for men. CDC defines binge drinking as consuming 4 or more drinks during 1 occasion in the last month for women and consuming 5 or more drinks during 1 occasion in the last month for men.

**Protective health practices. **Receiving a health checkup was defined as having had a routine checkup with a doctor in the past 2 years. Insurance status classified people into those with or without current health insurance. Influenza immunization included those who had received an influenza shot or spray, mist, or drop in the nose in the past year. Whether participants met recommendations for fruits and vegetables consumption and physical activity was based on CDC recommendations appropriate for the period of this study (11). Other measures included respondents who had their teeth cleaned in the last 12 months, had ever been tested for HIV, and had their cholesterol checked in the past 5 years.

### Data analyses

Analyses were conducted using the Stata statistical software (StataCorp LP; version 11), and we established significance at a .05 level. Pearson χ^2^ test for independence was used to determine whether demographic variables significantly differed by sexual orientation and sex. Demographic variables that were significant were assessed as independent variables for logistic regression models. Independent variables for the final logistic regression models were chosen using the backward elimination variable selection process. Age, education, relationship status, and urban or rural residency were included in the final models. Rural or urban residency was not a significant contributor in some of the final models; however, previous literature has established this variable as associated with health outcomes among LGB people (12,13). Although employment status and household income were each significant for women in bivariate analyses, they were not included for multivariable adjustment because they did not significantly contribute to the final models.

## Results

A total of 16,475 men and 26,271 women respondents were included in the analysis. Of these respondents, 268 men self-identified as gay and 123 as bisexual; 347 women self-identified as lesbian and 322 as bisexual.

### Demographics

Among men, 1.6% reported being gay and 0.7% bisexual; among women, 1.3% reported being lesbian and 1.2% bisexual. Individuals who reported being gay, lesbian, or bisexual were younger than heterosexual men and women; bisexuals were youngest in each sex group (Table 1). Race and ethnicity were not associated with sexual orientation in either men or women. Relationship status, educational attainment, and living in an urban or rural area were significantly different by sexual orientation. More heterosexuals were married or members of an unmarried couple compared with gay men, lesbian women, and bisexual men and women. Among men, gay men were the most educated, and bisexual and heterosexual men had similar levels of educational attainment. Among women, lesbian women were the most educated, followed by bisexual, and then heterosexual women. Gay men, lesbian women, and bisexual men and women were more likely to live in urban areas compared with their heterosexual counterparts. Employment status and household income were significantly different by sexual orientation for women, but not for men. Lesbian women were more likely to be employed compared with bisexual and heterosexual women, and lesbian and heterosexual women had a higher household income than bisexual women.

**Table 1:** Characteristics of Study Population, by Sex and Sexual Orientation, Oregon Behavioral Risk Factor Surveillance System, 2005–2008

Outcome	Men				Women			
	Weighted %			*P* a	Weighted %			*P* a
	Heterosexual	Gay	Bisexual		Heterosexual	Lesbian	Bisexual	
**Age, y**								
18–34	31.8	29.3	45.1	.003	28.8	26.9	62.3	<.001
35–54	38.1	50.0	33.1		36.9	54.4	30.3	
≥55	30.1	20.8	21.8		34.3	18.7	7.3	
**Relationship status**								
Married or a member of an unmarried couple	69.4	42.3	45.0	<.001	66.3	51.6	52.4	<.001
Formerly married	11.8	10.7	15.8		21.3	21.3	19.0	
Never married	18.8	47.0	39.3		12.4	27.1	28.6	
**Education**								
≤High school graduate or GED	36.9	23.7	36.0	.02	36.3	19.1	27.9	<.001
1–3 college years	27.2	28.8	28.7		31.7	27.3	33.7	
≥4 college years	35.9	47.4	35.3		32.0	53.6	38.4	
**Residency^b^**								
Urban	69.4	79.2	78.6	.03	69.3	77.3	78.4	.01
Rural	30.6	20.8	21.4		30.7	22.7	21.6	
**Employment status**								
Employed	66.6	74.9	72.3	.09	51.0	73.9	57.0	<.001
Unemployed or unable to work	10.8	9.5	9.6		9.5	13.1	15.0	
Not in workforce	22.6	15.6	18.1		39.5	13.0	28.0	
**Annual household income, $**								
<20,000	11.1	11.0	17.0	.32	14.2	13.0	20.4	<.001
20,000–49,999	42.9	46.1	47.1		44.9	46.6	55.8	
≥50,000	46.0	42.9	35.9		40.8	40.4	23.8	
**Race/ethnicity**								
White, non-Latino	85.9	88.8	83.4	.51	86.7	81.6	85.8	.14
All other races and Latino ethnicity	14.1	11.2	16.6		13.3	18.4	14.2	

Abbreviation: GED, general educational development.

a Calculated using a Pearson χ^2^ test.

b Rural or urban residency was determined using zip codes classified by Rural-Urban Commuting Area codes (10).

### Chronic conditions and health limitations

Gay and heterosexual men were not significantly different with respect to the chronic conditions and health limitations assessed in the study (Table 2). Bisexual men were significantly more likely to have a disability that limits activities compared with heterosexual men. Lesbian women were significantly more likely to have been diagnosed with arthritis and to have a disability that limits activities compared with heterosexual women. Compared with heterosexual women, bisexual women were significantly more likely to have poor mental or physical health, asthma, a disability that limits activities, and a disability that requires the use of special equipment.

**Table 2:** Prevalence of Chronic Conditions and Health Limitations and the Association with Sexual Orientation, by Sex, Oregon Behavioral Risk Factor Surveillance System, 2005–2008

Characteristic	Men			Women		
	Weighted % (95% CI)	AOR^a^ (95% CI)	*P* b	Weighted % (95% CI)	AOR^a^ (95% CI)	*P* b
**Mental or physical health not good (on ≥7 days during the past 30 days)**						
Heterosexual (Ref)	16.5 (15.4–17.7)	—	—	17.3 (16.5–18.2)	—	—
Gay or lesbian	15.4 (9.9–23.1)	0.8 (0.5–1.4)	.47	15.9 (10.9–22.6)	1.0 (0.6–1.6)	.96
Bisexual	17.2 (9.1–29.9)	1.1 (0.6–2.4)	.73	24.6 (18.0–32.6)	2.0 (1.3–3.1)	<.001
**Had cardiovascular disease^c^**						
Heterosexual (Ref)	8.0 (7.5–8.7)	—	—	6.2 (5.8–6.6)	—	—
Gay or lesbian	6.0 (3.2–10.8)	0.9 (0.5–1.7)	.73	4.0 (2.1–7.5)	1.0 (0.5–1.9)	.88
Bisexual	11.4 (5.8–21.1)	1.8 (0.8–3.7)	.12	1.8 (0.6–6.0)	0.7 (0.2–2.9)	.61
**Had high blood pressure**						
Heterosexual (Ref)	27.2 (25.6–28.9)	—	—	25.6 (24.3–26.8)	—	—
Gay or lesbian	17.9 (10.7–28.2)	0.6 (0.4–1.1)	.09	22.9 (13.8–35.7)	1.2 (0.6–2.4)	.66
Bisexual	16.1 (8.0–29.8)^d^	0.5 (0.2–1.1)	.09	12.4 (7.5–19.9)	0.9 (0.5–1.7)	.85
**Had high cholesterol**						
Heterosexual (Ref)	40.2 (38.2–42.2)	—	—	36.6 (35.1–38.1)	—	—
Gay or lesbian	33.3 (20.2–49.8)	1.0 (0.5–1.7)	.88	42.2 (29.2–56.5)	1.6 (0.8–3.3)	.19
Bisexual	37.7 (18.5–61.6)^d^	0.8 (0.3–2.0)	.62	22.5 (13.5–35.2)	0.9 (0.5–1.7)	.81
**Had diabetes**						
Heterosexual (Ref)	6.9 (6.4–7.4)	—	—	6.5 (6.1–6.8)	—	—
Gay or lesbian	7.8 (4.4–13.5)	1.3 (0.7–2.5)	.42	10.8 (4.0–26.0)^d^	2.2 (0.6–7.8)	.22
Bisexual	2.3 (0.7–7.0)	0.4 (0.1–1.1)	.08	2.4 (1.2–5.0)^d^	0.8 (0.4–1.6)	.50
**Had asthma**						
Heterosexual (Ref)	7.0 (6.4–7.6)	—	—	12.1 (11.5–12.7)	—	—
Gay or lesbian	9.2 (5.9–14.1)	1.4 (0.8–2.3)	.20	15.4 (10.8–21.7)	1.2 (0.8–1.9)	.37
Bisexual	8.5 (4.1–16.8)	0.9 (0.4–2.0)	.84	25.6 (18.6–34.2)	2.4 (1.5–3.6)	<.001
**Had arthritis**						
Heterosexual (Ref)	21.8 (20.5–23.0)	—	—	31.4 (30.3–32.6)	—	—
Gay or lesbian	26.3 (17.0–38.3)	1.5 (0.8–2.6)	.21	42.9 (32.5–53.9)	2.0 (1.2–3.3)	.005
Bisexual	14.9 (6.7–29.8)	0.9 (0.4–1.9)	.71	21.4 (14.0–31.2)	1.4 (0.8–2.6)	.30
**Had a disability (limited in activities)**						
Heterosexual (Ref)	21.6 (20.8–22.4)	—	—	24.8 (24.1–25.4)	—	—
Gay or lesbian	26.9 (20.6–34.2)	1.3 (0.9–1.9)	.23	36.6 (29.6–44.3)	1.9 (1.3–2.6)	<.001
Bisexual	29.6 (20.7–40.3)	1.6 (1.0–2.7)	.049	34.5 (28.1–41.6)	2.3 (1.7–3.2)	<.001
**Had a disability (need special equipment)**						
Heterosexual (Ref)	6.8 (6.4–7.3)	—	—	7.8 (7.4–8.1)	—	—
Gay or lesbian	6.0 (3.6–9.8)	0.8 (0.4–1.3)	.36	10.0 (4.7–20.0)	1.5 (0.6–3.8)	.42
Bisexual	10.8 (6.2–18.2)	1.8 (1.0–3.3)	.07	6.4 (4.3–9.6)	1.7 (1.1–2.7)	.03

Abbreviations: CI, confidence interval; AOR, adjusted odds ratio; Ref, reference variable; — , not applicable.

a Odds ratio is adjusted for age, education, relationship status, and urban or rural residency.

b Calculated using logic regression modeling.

c Cardiovascular disease included respondents who had ever had a heart attack, angina or coronary heart disease, or a stroke.

d This number’s coefficient of variance was greater than 30% and therefore may be statistically unreliable and should be interpreted with caution.

### Health risk factors and protective health practices

Compared with heterosexual men, gay men were significantly less likely to be obese and significantly more likely to binge drink (Table 3). Bisexual men were significantly more likely to be current cigarette smokers and to binge drink compared with heterosexual men. Lesbian and bisexual women were significantly more likely to be current cigarette smokers and to be obese compared with heterosexual women. Bisexual women were also significantly more likely to binge drink compared with heterosexual women, but lesbian women were not (*P* = .054). However, because the significance level for binge drinking among lesbian women was borderline, we included them in the discussion as significant.

**Table 3:** Prevalence of Health Risk Factors and Protective Health Practices and the Association With Sexual Orientation, by Sex, Oregon Behavioral Risk Factor Surveillance System, 2005–2008

Characteristic	Men			Women		
	Weighted % (95% CI)	AOR^a^ (95% CI)	*P* b	Weighted % (95% CI)	AOR^a^ (95% CI)	*P* b
**Health risk factors^c^**						
**Current cigarette smoker**						
Heterosexual (Ref)	18.6 (17.7–19.4)	—	—	15.3 (14.7–15.9)	—	—
Gay or lesbian	22.9 (16.9–30.1)	1.2 (0.8–1.8)	.29	22.5 (15.7–31.1)	1.6 (1.1–2.3)	.02
Bisexual	31.4 (21.2–43.6)	1.9 (1.1–3.2)	.02	37.3 (29.3–43.9)	2.8 (2.0–3.9)	<.001
**Obese**						
Heterosexual (Ref)	25.2 (24.4–26.1)	—	—	24.2 (23.5–24.9)	—	—
Gay or lesbian	15.7 (11.3–21.4)	0.6 (0.4–0.9)	.01	32.8 (26.9–39.3)	1.6 (1.2–2.1)	.002
Bisexual	22.0 (14.4–32.1)	0.9 (0.5–1.5)	.68	33.7 (24.8–42.4)	1.7 (1.2–2.4)	.001
**Binge drinker**						
Heterosexual (Ref)	18.7 (17.6–19.9)	—	—	8.9 (8.2–9.6)	—	—
Gay or lesbian	31.9 (22.0–43.8)	1.8 (1.1–3.1)	.02	16.4 (10.4–25.0)	1.7 (1.0–3.0)	.054
Bisexual	34.2 (21.3–49.9)	2.1 (1.0–4.2)	.04	25.4 (17.2–35.8)	2.5 (1.5–4.1)	.001
**Heavy drinker**						
Heterosexual (Ref)	5.5 (4.9–6.2)	—	—	5.6 (5.1–6.2)	—	—
Gay or lesbian	10.2 (5.3–18.7)	1.8 (0.9–3.7)	.13	8.5 (4.7–14.9)	1.4 (0.7–2.7)	.31
Bisexual	4.4 (1.6–11.6)	0.7 (0.2–2.1)	.54	7.1 (3.8–13.0)	1.3 (0.6–2.5)	.51
**Protective health practices**						
**Had health insurance^d^**						
Heterosexual (Ref)	82.6 (81.6–83.6)	—	—	87.1 (86.3–87.7)	—	—
Gay or lesbian	79.6 (71.9–85.7)	0.8 (0.5–1.3)	.41	91.1 (86.9–94.1)	1.5 (0.9–2.4)	.10
Bisexual	80.5 (67.0–89.4)	1.3 (0.6–2.6)	.52	71.4 (62.6–78.9)	0.5 (0.3–0.7)	.001
**Had health checkup in past 2 years**						
Heterosexual (Ref)	70.6 (69.6–71.6)	—	—	82.2 (81.5–82.9)	—	—
Gay or lesbian	69.9 (61.6–77.0)	1.0 (0.7–1.5)	.93	78.0 (69.4–84.8)	0.8 (0.6–1.3)	.40
Bisexual	64.3 (52.4–74.6)	0.8 (0.5–1.3)	.40	66.3 (58.9–73.1)	0.5 (0.4–0.7)	<.001
**Got influenza immunization**						
Heterosexual (Ref)	28.9 (27.8–30.0)	—	—	36.2 (35.3–37.2)	—	—
Gay or lesbian	42.9 (33.4–53.0)	2.5 (1.5–4.0)	<.001	33.5 (24.1–44.5)	1.1 (0.6–2.0)	.69
Bisexual	26.6 (15.6–41.6)	1.1 (0.5–2.4)	.82	22.8 (15.0–33.1)	0.9 (0.5–1.6)	.70
**Ever tested for HIV**						
Heterosexual (Ref)	33.6 (32.1–35.1)	—	—	37.6 (36.3–38.8)	—	—
Gay or lesbian	73.4 (60.9–83.1)	5.4 (3.0–9.7)	<.001	45.9 (36.9–55.2)	1.3 (0.9–2.0)	.14
Bisexual	67.0 (49.6–80.7)	4.1 (1.9–8.8)	<.001	66.3 (56.2–75.1)	2.6 (1.7–4.2)	<.001
**Checked cholesterol in past 5 years**						
Heterosexual (Ref)	67.4 (65.3–69.5)	—	—	72.9 (71.1–74.6)	—	—
Gay or lesbian	79.3 (66.5–88.1)	2.0 (0.9–4.7)	.10	75.5 (58.0–87.3)	1.1 (0.6–2.1)	.80
Bisexual	65.0 (43.3–81.9)	0.9 (0.5–1.8)	.77	62.7 (48.5–74.9)	1.5 (0.6–3.5)	.37
**Met CDC fruit and vegetable recommendations**						
Heterosexual (Ref)	20.4 (19.1–21.9)	—	—	32.3 (31.0–33.6)	—	—
Gay or lesbian	26.1 (16.3–39.0)	1.4 (0.8–2.6)	.23	31.0 (22.3–41.2)	0.9 (0.6–1.4)	.58
Bisexual	28.0 (14.7–46.7)	1.6 (0.7–3.7)	.27	35.5 (24.2–48.7)	1.1 (0.6–2.1)	.68
**Met CDC physical activity recommendations**						
Heterosexual (Ref)	57.4 (55.7–59.2)	—	—	55.7 (54.3–57.1)	—	—
Gay or lesbian	57.8 (45.5–69.3)	1.0 (0.6–1.6)	.91	50.4 (39.5–61.2)	0.8 (0.5–1.2)	.24
Bisexual	59.8 (39.6–77.1)	1.0 (0.5–2.3)	.96	53.7 (41.4–65.6)	0.8 (0.5–1.3)	.35
**Had teeth cleaned in past 12 months**						
Heterosexual (Ref)	66.0 (64.3–67.7)	—	—	68.9 (67.6–70.2)	—	—
Gay or lesbian	68.9 (54.1–80.6)	1.2 (0.6–2.4)	.64	59.5 (48.4–69.8)	0.5 (0.3–0.9)	.01
Bisexual	48.9 (29.7–68.5)	0.5 (0.2–1.1)	.09	43.3 (30.9–56.6)	0.4 (0.2–0.6)	<.001

Abbreviation: CI, confidence interval; AOR, adjusted odds ratio; Ref, reference variable; — , not applicable; HIV, human immunodeficiency virus; CDC, Centers for Disease Control and Prevention.

a Odds ratio is adjusted for age, education, relationship status, and urban or rural residency.

b Calculated using logic regression modeling.

c Current cigarette smokers were defined as having smoked 100 or more cigarettes in their lifetime and currently smoking cigarettes on some or all days. Obesity was established for respondents who had a body mass index (weight in kilograms divided by the square of height in meters) of 30 kg/m^2^ or higher. CDC defines heavy drinking as consuming more than 1 drink per day on average in the last month for women and consuming more than 2 drinks per day on average in the last month for men. CDC defines binge drinking as consuming 4 or more drinks during 1 occasion in the last month for women and consuming 5 or more drinks during 1 occasion in the last month for men.

d Data were not available in 2008.

Compared with heterosexual men, gay men were significantly more likely to have received an influenza immunization and ever have been tested for HIV (Table 3). Bisexual men were significantly more likely to ever have been tested for HIV compared with heterosexual men. Compared with heterosexual women, lesbian women were significantly less likely to have had their teeth cleaned in the past 12 months. Bisexual women were significantly less likely to have health insurance, to have had a health checkup in the last 2 years, and to have had their teeth cleaned in the past 12 months and were more likely to ever have been tested for HIV compared with heterosexual women.

## Discussion

This study confirms many LGB health disparities found in prior studies and expands upon the chronic conditions, health risk factors, and protective health practices previously investigated using BRFSS data. New to the literature are results related to arthritis, obese weight status separate from overweight status, binge drinking, cholesterol screening, receiving a health checkup, HIV testing practices, and dental care. Although a previous study of LGB health controlled for age and education (4), our study additionally controls for the effects of relationship status and rural or urban residency. We found that the LGB population was less likely to be married or a member of an unmarried couple and more likely to live in an urban area. Marriage provides economic and health benefits (14,15), and rural and urban residency confers different health disparities, particularly in the extremely rural or urban areas (12,13). Cherlin hypothesizes that same-sex unmarried couples occupy a middle position, reporting better health than different-sex unmarried couples but poorer health than married couples (16).

Consistent with an analysis of health outcomes among LGB individuals in Washington State (4), this study shows that lesbian and bisexual women have more elevated health risks than heterosexual women. In contrast, gay or bisexual men are at elevated odds for only 3 of the health issues assessed, and gay men engage in more protective health practices compared with heterosexual men. It is not known why bisexual women have worse health outcomes than other groups. Dilley et al (4) hypothesize that bisexual orientation may contribute to negative health outcomes because of the stress associated with self-perceptions that shift between heterosexuality and homosexuality.

Lesbian women and bisexual men and women have a significantly higher prevalence of current cigarette smoking, lesbian and bisexual women have a significantly higher prevalence of obesity, and all LGB groups have a significantly higher prevalence of binge drinking. These behaviors and conditions are all leading preventable causes of premature death in the United States (17,18). The decreased likelihood of being obese among gay and bisexual men could be a result of high body dissatisfaction (19). In contrast, a recent study suggested that lesbian and bisexual women have higher rates of body satisfaction and higher ideal weights compared with heterosexual women (20), which could explain higher rates of obesity. However, another study found no such difference (21). Our study indicates that cigarette smoking among gay men is higher than among heterosexual men, although not significantly higher. Other studies show significantly higher cigarette smoking prevalence in the gay community (3,4). It is unknown why this study’s results differed, and more research is warranted. We also found binge drinking to be significantly higher among all sexual orientation minority groups compared with heterosexual men and women. This finding confirms those of a Massachusetts study that reported higher binge drinking among bisexual women compared with heterosexual women; however, the Massachusetts study did not find the same disparity among gay and bisexual men or among lesbian women (3).

LGB individuals did not differ from their heterosexual counterparts with respect to most of the chronic conditions assessed. This is likely because LGB men and women in this study were younger than heterosexual men and women and therefore less likely to have chronic conditions often associated with older age. However, the LGB groups were more likely to report risk factors associated with developing chronic conditions later in life. Although gay men in this study were less likely to be obese and more likely to engage in protective health behaviors, they were also more likely to binge drink and, although not seen in this study, previous literature has consistently found that gay men were significantly more likely to smoke cigarettes compared with heterosexual men (3,4). A recent study confirms that older LGB adults continue to be disproportionately affected by chronic disease risk factors, including cigarette smoking and excessive drinking (22), and are more likely to report chronic conditions associated with these risk factors, including cardiovascular disease among lesbian and bisexual women and poor physical health among gay and bisexual men (22).

Disparate engagement in unhealthy behaviors is also observed in other vulnerable populations, including those with low socioeconomic status (23,24). However, gay men and lesbian women in this study have higher educational attainment and are more likely to be employed than heterosexual men and women. A possible explanation is minority stress. The excessive and chronic stress to which LGB individuals are exposed as a result of stigmatization may lead to unhealthy coping mechanisms regardless of their higher educational attainment and employment (25,26). Although there are few best or promising practices for targeted public health policy and systems changes that address these disparities for LGB individuals, research suggests that systems-level policies that reduce stigma and systematic discrimination should reduce unhealthy behaviors (27,28). A study by Hatzenbuehler et al found an increase in mood disorders, generalized anxiety disorders, alcohol use, and psychiatric comorbidity over time among LGB people living in states with gay marriage bans, which was not observed among LGB people living in states without bans (29). In contrast, gay men in legally recognized civil unions have significantly greater social and emotional support and better access to health insurance and quality health care (15). A study in Massachusetts reported significant decreases in medical and mental health care visits and costs among gay men in the 12-month period after the enactment of a law granting marriage equality (30). One possible conclusion is that any public health interventions targeted directly to the LGB community may be ineffective if delivered in a climate of systematic discrimination.

This study had several limitations. The BRFSS sampling methods used during the period of this study excluded people living in homes without a landline telephone, those with only cellular telephone service, those living in institutions, and those who do not speak English or Spanish. Because of the small sample size, we were unable to adjust for race and ethnicity in the analysis and were unable to analyze depression among LGB people. Another limitation of this study was that sexual orientation was self-reported. Some LGB individuals may have been unwilling to describe themselves as gay, lesbian, or bisexual on a telephone survey. Dilley et al provide additional details on the limitations of the BRFSS for analysis of the LGB population (4). Future studies could include race and ethnicity, legal domestic partnership and marriage, and a more granular gradient of rural and urban residency as potential adjustment factors, and could include mental health conditions as a dependent variable in analysis.

We found disparities in chronic conditions and health limitations, health risk factors, and protective health practices among LGB men and women. These disparities were most prominent among lesbian and bisexual women, and gay men had the most protective health practices. However, compared with heterosexual men, gay men were more likely to engage in risky behaviors that lead to chronic diseases later in life, and lower prevalence of obesity is likely a result of negative body image. We confirm some of the LGB health disparities identified in previous studies and find additional areas to target tailored public health interventions. Stigma and discrimination may increase LGB individuals’ engagement in unhealthy behaviors, such as tobacco and alcohol use. Targeted public health interventions are likely most effective at improving health outcomes for LGB individuals if they are provided in systems that avoid stigmatizing and discriminating against LGB individuals where they live, work, learn, and socialize.
